# Repellent Activity of the Botanical Compounds Thymol, Carvacrol, Nootkatone, and Eugenol Against *Amblyomma sculptum* Nymphs

**DOI:** 10.3390/pathogens14090926

**Published:** 2025-09-13

**Authors:** Mayara Macêdo Barrozo, Emilly Faria Santos, Haile Dean Figueiredo Chagas, Rafael Assunção Carvalho, Isabela Santos Silva, Ariel de Souza Oliveira, Laura Cristina Ferreira Faria, Ana Lúcia Coutinho Teixeira, Viviane Zeringota, Hermes Ribeiro Luz, Lorena Lopes Ferreira, Caio Monteiro

**Affiliations:** 1 Centro de Parasitologia Veterinária, Escola de Veterinária e Zootecnia, Universidade Federal de Goiás, Av. Esperança, s/n, Campus Samambaia, Goiânia 74690-900, Brazil; emillyfaria@discente.ufg.br (E.F.S.); haile.dean@discente.ufg.br (H.D.F.C.); rafaelassuncao@discente.ufg.br (R.A.C.); isabelasantoscbio@gmail.com (I.S.S.); claura@discente.ufg.br (L.C.F.F.); luciacoutinho13@gmail.com (A.L.C.T.); caiosat@gmail.com (C.M.); 2 Instituto de Patologia Tropical e Saúde Pública, Universidade Federal de Goiás, Rua 235 s/n, Setor Universitário, Goiânia 74605-050, Brazil; arielsouza@discente.ufg.br (A.d.S.O.); viviane_zeringota@ufg.br (V.Z.); 3 Departamento de Patologia, Programa de Pós-Graduação em Biotecnologia do Renorbio, Ponto Focal Maranhão, Universidade Federal do Maranhão, São Luís 65085-580, Brazil; hermes.luz@ufma.br; 4 Departamento de Medicina Veterinária Prevntiva, Escola de Veterinária, Universidade Federal de Minas Gerais, Av. Antônio Carlos 6627 Caixa Postal 567, Campus Pampulha, Belo Horizonte 31270-901, Brazil; loren4_lopes@hotmail.com

**Keywords:** *Amblyomma cajennense* complex, DEET, essential oil, phenylpropanoid, terpene

## Abstract

This study evaluated the repellent activity of the botanical compounds thymol, carvacrol, nootkatone, and eugenol (5%), as well as the combination of 2.5% nootkatone + 2.5% eugenol, against *Amblyomma sculptum* nymphs under laboratory and field conditions. In contact bioassays, carvacrol and nootkatone showed the highest mean repellency rates (94.5% and 93.7%), followed by thymol and eugenol (90.2% and 87.2%). The combination (nootkatone + eugenol) resulted in 92.8% repellency, with 100% efficacy in some evaluation periods. The repellency of 7% DEET (positive control) was 82.2%. Nootkatone 5% and the combination (nootkatone + eugenol) were also tested in a Y-tube olfactometer, showing repellency rates of 86.1% and 72.2%, respectively, both higher than 7% DEET (69.4%). In field trials, volunteers wore treated socks and walked through an area naturally infested with nymphs. A significant reduction (*p* < 0.05) in tick counts was observed in the treated groups (about five unfed nymphs) compared to the control (about 40 unfed nymphs). Cumulative efficacy exceeded 85% in both treatments. In the cost simulation, the estimated cost of a 100 mL formulation containing 5% nootkatone was USD 50.8, while the combination (nootkatone + eugenol) presented a cost USD 28.6, representing a 44% reduction with no loss of efficacy in field conditions. These results indicate that all compounds tested showed repellent activity in the laboratory. Nootkatone has high repellent activity, and its combination with eugenol is a promising and more economically viable alternative for tick repellency.

## 1. Introduction

Tick-borne pathogens have become an increasing public health concern in the Americas, particularly *Spotted Fever*, a severe zoonosis caused by the bacterium *Rickettsia rickettsii*, which, if not treated promptly, presents high lethality in humans. The tick *Amblyomma sculptum*, found in several regions of Brazil and in other South American countries such as Bolivia, Paraguay, and Argentina, is considered the main vector of this bacterium to humans in the country. Horses (*Equus caballus*), capybaras (*Hydrochoerus hydrochaeris*), and tapirs (*Tapirus terrestris*) are its primary hosts of *A. sculptum*; however, due to its low host specificity, this tick can be found parasitizing a wide range of mammalian species, including humans. Notably, *A. sculptum* is considered the main tick species parasitizing humans in Brazil [1,2,3,4,5].

Topical repellents, such as N,N-diethyl-3-methylbenzamide (DEET), which is considered the gold standard against arthropods (including ticks), and icaridin, to a lesser extent, are used to prevent tick bites and, consequently, the transmission of pathogens [6,7,8]. Although DEET is widely used effectively and is generally considered safe for users, some reports of adverse effects, especially in children, pregnant women, and the elderly, as well as concerns about toxicity from prolonged exposure, have encouraged the search for natural compounds as safer alternatives [9,10]. In this context, interest in botanically derived compounds has increased due to potential advantages such as biodegradability, lower toxicity, and reduced environmental impact [6,11,12,13]. Among the promising compounds for use as tick repellents are the phenolic monoterpenes thymol and carvacrol, the sesquiterpene nootkatone, and the phenylpropanoid eugenol [14,15,16,17,18].

Thymol and carvacrol are terpenes found in essential oils (EOs) of *Thymus vulgaris*, *Origanum vulgare*, and *Lippia* spp., which have demonstrated repellent activity against different tick species [19,20,21]. Nootkatone, found in the essential oil of *Chamaecyparis nootkatensis* (Alaska yellow cedar) and *Citrus* × *paradisi* (grapefruit), was registered in 2020 by the United States Environmental Protection Agency (EPA) as a natural repellent and pesticide [22]. Laboratory and field studies have demonstrated its high efficacy against different tick species [23,24,25]. Eugenol, in turn, is the major compound in the essential oil of *Syzygium aromaticum* (clove) and exhibits multiple biological activities, such as antifungal, antimicrobial, insecticidal, acaricidal, and repellent effects, including against ticks [26,27,28,29]. However, one of the main challenges for practical application, especially of nootkatone, is its high cost, which may compromise the economic feasibility of commercial formulations [23,25,30,31]. Therefore, it becomes relevant and desirable to explore combinations of nootkatone with other, lower-cost molecules that can enhance its repellent activity, enabling the use of lower concentrations without compromising efficacy.

Despite recent advances, the inclusion of natural compounds in commercial repellent formulations remains limited [6]. Further scientific evidence is needed to confirm their spatial and contact repellent efficacy in both laboratory and field conditions, supporting the development of safer and more environmentally sustainable strategies to reduce human exposure to ticks. Thus, this study aimed to evaluate the repellent activity of thymol, carvacrol, nootkatone, and eugenol against *A. sculptum* nymphs using three complementary approaches, including two distinct laboratory bioassays: Petri dishes (one-choice test) to assess contact repellency, including both isolated compounds and binary combinations (nootkatone + eugenol) and olfactory bioassays (olfactometer) to assess spatial repellency of nootkatone (alone or in combination with eugenol), and a field trial.

## 2. Material and Methods

### 2.1. Ticks

Engorged females of *A. sculptum* were collected from naturally infested cattle at the School of Veterinary and Animal Science (EVZ), Federal University of Goiás (UFG), in Goiânia, Brazil. After collection, the females were incubated under controlled temperature (27 ± 1 °C) and relative humidity (85 ± 5%) conditions for oviposition and larval hatching. *Amblyomma sculptum* larvae were fed on California × New Zealand rabbits (*Oryctolagus cuniculus*) to obtain engorged larvae. These larvae were then incubated under the same conditions described above to obtain unfed nymphs. The protocol was approved by the Animal Ethics Committee of UFG (CEUA-UFG, no. 053/21). Unfed nymphs, aged between 15 and 60 days, were selected for the laboratory repellency bioassays, as they represent the stage with the highest competence in the transmission of *R. rickettsii* to humans [32].

### 2.2. Test Compounds

Thymol, carvacrol, nootkatone, eugenol, and DEET were purchased from Sigma-Aldrich^®^ with purity ≥ 98%. In the Petri dish laboratory tests with unfed *A. sculptum* nymphs, the botanical compounds were applied individually at a concentration of 5%, while DEET was used at a concentration of 7% as a positive repellency control. The binary combination of nootkatone and eugenol was tested at a ratio of 2.5 + 2.5%, aiming to reduce the concentration of nootkatone by half and therefore its cost, while maintaining efficacy. Ethanol (Neon^®^; 50% *v*/*v* in water—hydroethanolic) was used as the solvent to prepare test solutions for both laboratory and field assays. The choice of ethanol was based on its low toxicity to *A. sculptum*, as documented in the literature [33,34]. The compounds that showed the best results in contact tests, along with greater viability for practical application, were selected for further evaluation in olfactometer and field assays.

### 2.3. Laboratory Bioassays

#### 2.3.1. Contact Repellency in One-Choice Petri Dish Tests

**Figure 1:**
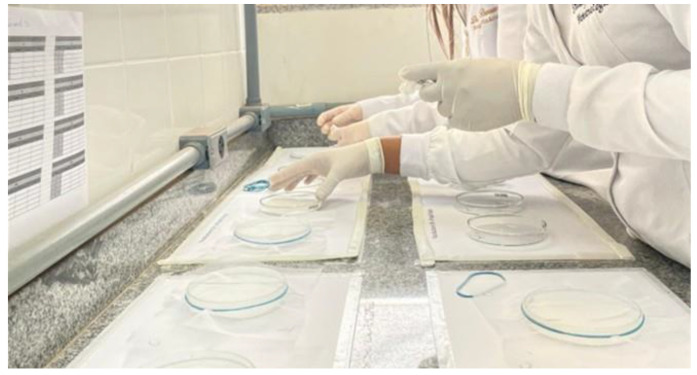
Repellency test in Petri dishes. The experiment was conducted on a laboratory bench, with each dish representing an experimental unit in which the ticks were exposed to the tested compounds.

Thymol, carvacrol, nootkatone, and eugenol, as well as the binary combination of nootkatone + eugenol, were subjected to repellency bioassays in Petri dishes following the methodology adapted from Barrozo et al. [34]. The experiments were conducted in a climate-controlled room at a temperature of 27 ± 1 °C and relative humidity of 70 ± 10%. The experimental “area preference method (arena)” consisted of 9 cm diameter glass Petri dishes containing two semicircles of filter paper (Whatman™, Cytiva, Maidstone, UK; cat. no. 1001), each with an area of 31.8 cm^2^, covering half of the bottom of the dish (Figure 1). One of the semicircles was treated with 150 μL of the test compound, while the other received 150 μL of 50% ethanol (solvent control—hydroethanolic solution). After application, the papers were left to dry for 10 min in a fume hood (Permution) before being placed in the dishes.

**Figure 2:**
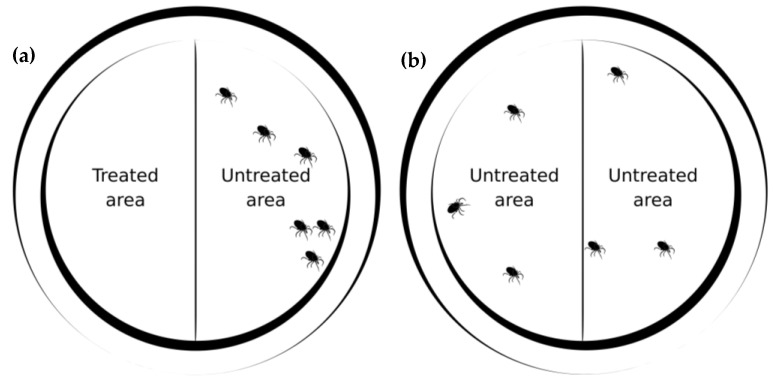
Schematic representation of the repellency test in Petri dishes. (**a**): Treated group, in which ticks tend to migrate toward the untreated semicircle, indicating repellent activity. (**b**): Control group with two untreated semicircles, in which ticks are expected to distribute randomly and evenly (approximately 50% on each side) across the filter paper surface. This is the expected behavior.

Six unfed *A. sculptum* nymphs were placed along the central line between the treated and control filter paper halves. To prevent the ticks from escaping, each dish was covered with a thin, transparent 100% polyamide fabric, previously treated with 150 μL of the same test solution on half of its surface (corresponding to the side above the treated semicircle) and allowed to dry for 10 min. After drying, the fabric was positioned over the dish and secured with an elastic band (Figure 2).

Two control groups were included to assess the potential influence of the solvent and filter paper on tick behavior. In the first group, one semicircle was treated with 50% hydroethanolic solution, while the other remained untreated (treated paper vs. untreated paper). In the second group, neither semicircle was treated (blank control: untreated paper vs. untreated paper). These controls were included to confirm that neither the paper nor the solvent influenced tick positioning, ensuring that any observed repellent effect was exclusively due to the presence of the botanical compounds (Figure 2).

The position of ticks on each plate was recorded at the following time points: 1, 15, and 30 min; and 1, 2, 3, 4, 24, 48, and 72 h after the start of the experiment. Ticks located on the untreated semicircle were considered repelled (Figure 2). The mean repellency percentage was calculated based on the proportion of ticks found on the control side of the plate. Experiments were conducted on two separate days, using five plates per day (each representing one experimental unit), totaling ten replicates. Compounds with greater practical application potential (i.e., greater safety for mammals) were selected for additional olfactometer assays to evaluate spatial repellency.

#### 2.3.2. Y-Tube Olfactometer Bioassay: Spatial Repellency

In this assay, nootkatone (5%), selected based on the Petri dish test, and the binary combination of nootkatone (2.5% *v*/*v*) + eugenol (2.5% *v*/*v*) were evaluated. A positive control group containing 7% DEET was also included. The Y-tube olfactometer bioassay was conducted following the methodology described by Ferreira et al. [35,36].

The test was conducted using a Y-tube arena made of acrylic (13 cm for the main stem, 18 cm for each arm, with a diameter of 6 cm), positioned vertically. At the opening of each arm (1 cm in diameter), a 50 mL glass jar, referred to as odor jar, was connected using silicone tubing. The airflow was generated by a vacuum pump (Diapump/Fanem) attached to the lower end of the main stem of the olfactometer through a silicone tube (2 m long and 1 cm in diameter). The flow rate was adjusted to 100 mL/min using a flow meter (Parker—P3A), and the air was previously filtered through activated charcoal (Fluka, Sigma–Aldrich), which was connected to the odor jar. For compound release, a piece of filter paper (1 cm × 4 cm, Whatman No. 1, qualitative) was treated with 11 μL of the test solution or the solvent (control), then dried under a fume hood for one minute before use. In each replicate, one arm received the airflow containing the odor of the test solution (5% nootkatone or 2.5% nootkatone + 2.5% eugenol), while the other arm received the odor of the solvent. A blank control was also included, in which a filter paper not treated was used, to ensure that the paper itself did not influence the ticks’ choice.

Nymphs were individually released at the base of the Y-tube olfactometer, and their choice was recorded over a period of two minutes. If the nymph did not make a choice, the individual was tested again for up to three attempts. If it still did not exhibit climbing behavior and choose one of the arms, the tick was excluded from the analysis. For each compound and concentration, 36 unfed nymphs were used. New treated filter papers were replaced every six minutes, and the position of the arms (treatment and control) was alternated with each paper change. The olfactometer was cleaned with 95% ethanol at the end of each testing day to avoid contamination.

### 2.4. Field Bioassays (Sock Assay)

The field study was conducted with the participation of 10 volunteers, including seven women and three men, who took turns randomly on the test days. All participants declared no allergy or sensitivity to the tested compounds. The project was approved by the Research Ethics Committee (CEP/UFG—No. 4.955.565).

Repellency tests were carried out at the Dairy Farm of EVZ/UFG, located in the municipality of Goiânia, state of Goiás, Brazil (16°36′24″ S; 49°15′34″ W). The region belongs to the Cerrado biome (tropical savanna), with a tropical “Aw” climate according to the Köppen–Geiger classification [37]. It features two well-defined seasons: a rainy season (October to April), with an average annual precipitation ranging from 1235 to 1424 mm, and a dry season (May to September), with precipitation between 150 and 200 mm. The local landscape consists of gently sloping plateaus, areas of pasture, scattered shrubs, and small forest fragments [38]. The area is inhabited by capybaras and cattle grazing on *Panicum maximum* pastures [39,40], and it presents a high density of *A. sculptum*. In a previous two-year study, 100,627 larvae, 10,055 nymphs, and 6977 adults were collected [39].

The methodology proposed by Bissinger et al. [41] and Barrozo et al. [38] was used. The experiments were conducted on three separate days between July and August 2022, the period of highest nymphal occurrence in the environment [5,39]. The tests were performed between 9:00 a.m. and 12:00 p.m., corresponding to the peak activity period for ticks in the study area. The study was carried out over three distinct days (three repetitions): on two days, three replicates were performed per treatment, and on one day, four replicates, totaling 10 replicates. The evaluation period was defined based on (i) a previous study documenting the seasonal dynamics of *A. sculptum* in the area [39] and (ii) field observations of peak nymph activity by the present research team.

**Figure 3:**
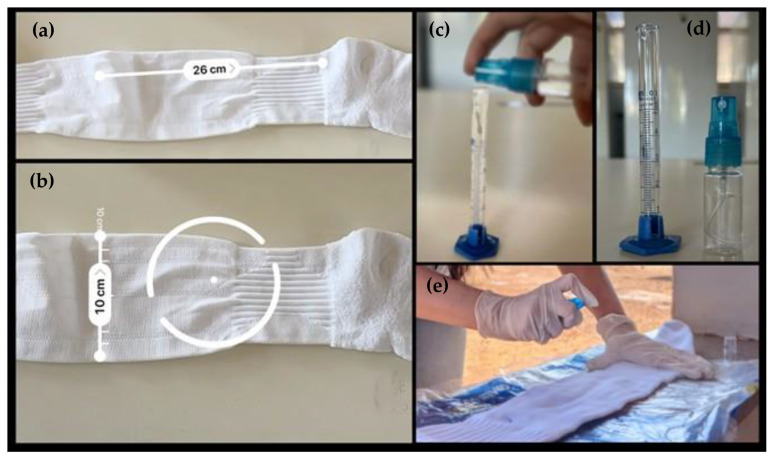
Preparation of the socks for the field repellency test. (**a**,**b**) Measurement of the external area of the sock to calculate the appropriate application volume (1 mL/600 cm^2^); (**c**,**d**) measurement of the solution volumes to be applied to the socks; (**e**) application of the solutions using a spray bottle on the sock surface.

Volunteers wore personal protective equipment (PPE), including white hooded coveralls sealed with adhesive tape at the sleeve ends and around the zippers. For the repellency assessment, participants wore Kanxa^®^ knee-high socks (49% polyamide, 34% cotton, 11% polyester, and 6% elastodiene) over the coveralls. The external surface of each sock was measured to determine the appropriate application volume (1 mL per 600 cm^2^). Repellent solutions were sprayed 15 min before testing, covering the sock area corresponding to the lower leg (Figure 3). Each sock was used only once. For each volunteer, one leg was treated with 5% nootkatone or the binary combination of 2.5% nootkatone + 2.5% eugenol, while the other leg received 50% ethanol (negative control).

**Figure 4:**
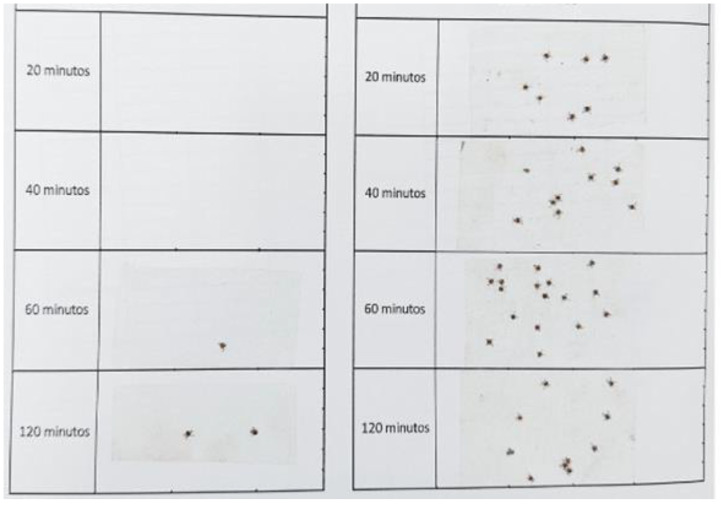
A4 sheet with adhesive tapes used to collect and fix *Amblyomma sculptum* nymphs that were not repelled and climbed onto the volunteers’ socks during field assays. Each column represents one leg, and the horizontal tape strips correspond to exposure times of 20, 40, 60, and 120 min.

Subsequently, volunteers walked predominantly along the forest edge, an area with a high density of *A. sculptum*, in a randomized order and at a slow pace (approximately 30 steps per minute) for 20 min during each evaluation period, covering approximately 80 m^2^. Each trial lasted a total of 120 min, with tick collections performed at 20, 40, 60, and 120 min. During the walk, ticks that climbed and remained on the socks were collected for later quantification and identification. To facilitate visualization and counting, the ticks were removed using transparent adhesive tape and fixed onto white paper labeled with the treatment group and evaluation period (Figure 4). Only nymphs were quantified and identified based on morphological criteria described by Martins et al. [42] and Suzin et al. [43].

The formula used to calculate repellency was as follows:

Repellency (%) = (C − T)/C × 100

where

C = number of nymphs collected from the control sock;

T = number of nymphs collected from the treated sock.

### 2.5. Cost Evaluation

For the cost evaluation, the prices of 100 mg of the compounds nootkatone and eugenol were considered, based on the values listed by Sigma-Aldrich (Merck KGaA, Darmstadt, Germany) [44,45] (accessed on 15 March 2025). Based on these prices, the estimated production cost for 100 mL of formulations containing (i) 5% nootkatone and (ii) 2.5% nootkatone + 2.5% eugenol was calculated. The volume of 100 mL was adopted as a reference, as it corresponds to the amount commonly used in commercially available repellent bottles. The calculation was performed by determining the quantity of each compound required to make up this volume, based on the cost per 100 mg provided by the supplier. The combined formulation was included to assess its economic feasibility by reducing the nootkatone concentration and overall cost while maintaining efficacy.

### 2.6. Data Analysis

Repellency results were expressed as mean ± standard deviation. The percentage of repellency for each compound and concentration was calculated based on the number of ticks located on the control side of the Petri dish or on the sock (treated or untreated).

For the comparison of tick distribution in the laboratory bioassays, the chi-square test was used, with the level of significance set at *p* < 0.05. In the olfactometer bioassay, the chi-square test was also applied to compare the choices of nymphs between the treated and control sides.

Statistical differences in field data were assessed using one-way analysis of variance (ANOVA) or Student’s *t*-test. Analyses were performed using GraphPad Prism software, version 5.03 (GraphPad Software, Inc., Boston, MA, USA).

## 3. Results

### 3.1. Contact Repellency in Petri Dish Tests

The results of the tests with isolated compounds and the binary combination are presented in Table 1. In the bioassays using 5% of thymol, carvacrol, nootkatone, and eugenol, significant repellency (*p* < 0.05) was observed at all evaluation time points throughout the 72-h test, except for thymol at 48 h. The positive control with 7% DEET also showed significant repellency at all evaluated time points (Table 1). The hydroethanolic solutions of nootkatone, carvacrol, and thymol exhibited mean repellency values above 90% after 72 h, while eugenol and DEET showed mean repellency of 87.2% and 82.2%, respectively (Table 1).

In the bioassays with the binary combination (nootkatone + eugenol), significant repellency (*p* < 0.05) was observed at all evaluation time points over the 72-h period, with percentages ranging from 83% to 100%. Notably, 100% repellency was recorded at the 2-, 3-, and 4-h evaluations, and the mean repellency after 72 h was 92.8% (Table 1).

As all treatments demonstrated high repellency, nootkatone and the combination of nootkatone + eugenol were selected for subsequent studies due to their greater practical applicability, because they have less potential for irritability/toxicity to mammals.

### 3.2. Spatial Repellency in Y-Tube Olfactometer

**Figure 5:**
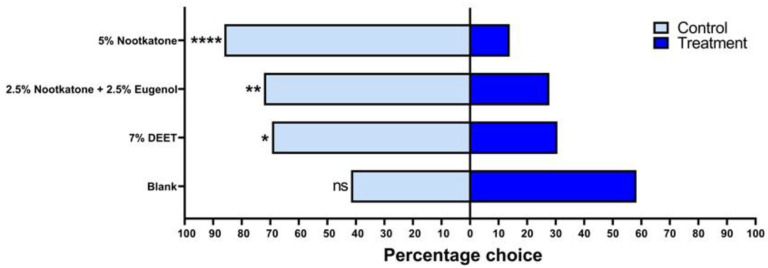
Repellency of unfed *Amblyomma sculptum* nymphs in Y-tube olfactometer assay with nootkatone (5%),the combination of nootkatone (2.5%) + eugenol (2.5%) and DEET (7%). Bars represent the mean percentage of ticks distributed between the control arm (light blue) and the treated arm (dark blue). Chi-square tests indicated significant differences for nootkatone (****), the combination of nootkatone + eugenol (**), and 7% DEET (*) at the 5% significance level. (* *p* < 0.05, ** *p* < 0.01, **** *p* < 0.0001, ns = not significant).

In the olfactory bioassay, nootkatone (5%) and the combination of nootkatone (2.5%) and eugenol (2.5%) were evaluated for their ability to spatially repel *A. sculptum* nymphs (Figure 5). When both odor jars were left with an untreated filter paper (control group), 41.7% of the nymphs migrated to the left arm and 58.3% to the right, indicating a random distribution (χ^2^ = 1; *p* = 0.31731). A repellency rate of 69.4% was observed for 7% DEET (7%) (χ^2^ = 5.444; *p* = 0.01963), while 5% nootkatone exhibited a higher repellency rate of 86.1% (χ^2^ = 18.778; *p* = 0.00002), indicating superior repellent activity compared to DEET (Figure 5). The binary combination of 2.5% nootkatone and 2.5% eugenol resulted in a repellency rate of 72.2%, with significant differences compared to both DEET and the control group (χ^2^ = 7.111; *p* = 0.00766) (Figure 5).

Due to these promising results, both nootkatone (5%) and the combination of nootkatone (2.5%) and eugenol (2.5%) were selected for field testing.

### 3.3. Field Bioassays

**Figure 6:**
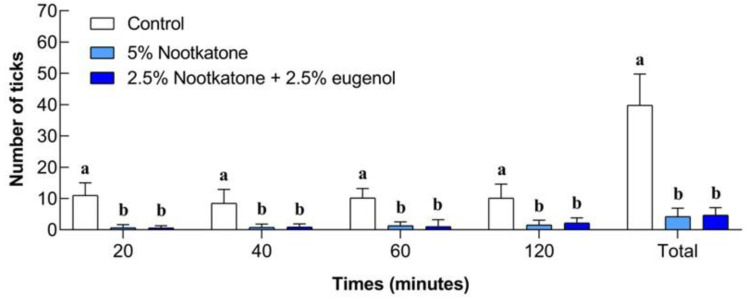
Number of *Amblyomma sculptum* nymphs collected while climbing on socks treated with 5% nootkatone or a combination of 2.5% nootkatone + 2.5% eugenol, and on untreated socks (control: 50% ethanol), at different evaluation times (20, 40, 60, and 120 min), during a field trial in a naturally infested area. Bars represent the mean ± standard deviation. Different letters indicate statistically significant differences between treatments at the same evaluation time (ANOVA, *p* < 0.05).

**Figure 7:**
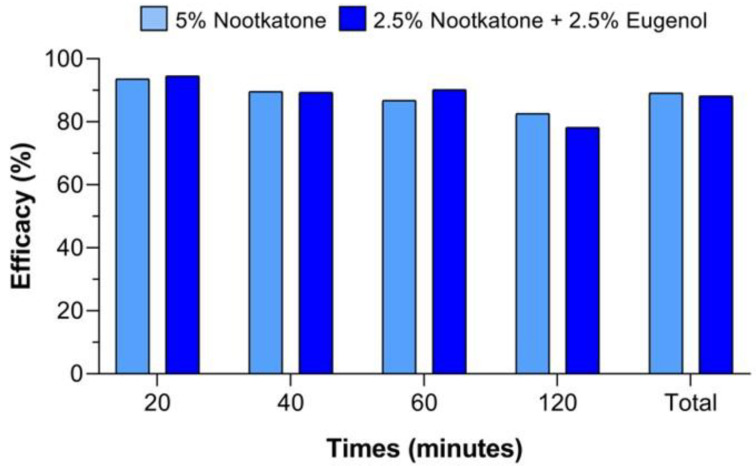
Repellent efficacy (%) observed in field trials (sock assay) using 5% nootkatone and the combination of 2.5% Nootkatone + 2.5% eugenol against unfed *Amblyomma sculptum* nymphs at different evaluation times (20, 40, 60, and 120 min) in a naturally infested area.

Field test results are shown in Figure 6 and Figure 7. All nymphs collected from the socks were identified as *A. sculptum*. The number of ticks collected from the treatments with nootkatone (5%) and the combination of nootkatone (2.5%) + eugenol (2.5%) was significantly lower (*p* < 0.05) than that collected from the control group at all time points evaluated (20, 40, 60, and 120 min). No significant differences (*p* > 0.05) were observed between the nootkatone and the binary combination treatments at any time point. The mean total number of ticks collected after 2 h was 39.8 in the control group, while the treated groups showed means of 4.2 (nootkatone) and 4.7 (nootkatone + eugenol) (Figure 6).

The repellent efficacy observed for nootkatone (5%) and the combination of nootkatone (2.5%) + eugenol (2.5%) remained above 80% across all evaluation periods, with cumulative efficacies of 89.1% and 87.6%, respectively (Figure 7).

### 3.4. Cost Assessment

Considering only the costs of active ingredients, the cost simulation for producing a 100 mL formulation containing 5% (*w*/*v*) nootkatone, the estimated cost was USD 50.8 (Table 2). For the formulation containing 2.5% nootkatone and 2.5% eugenol, the estimated cost was USD 28.6, representing a reduction of approximately 44% compared to the formulation with nootkatone alone. This cost reduction is noteworthy, as it increases the economic feasibility of using nootkatone as a tick repellent without compromising efficacy.

## 4. Discussion

In this study, we demonstrated that the botanical compounds nootkatone, carvacrol, thymol, and eugenol exhibit repellent activity in laboratory against *A. sculptum* nymphs. For the first time, it was shown that nootkatone (5%) and the binary combination of nootkatone (2.5%) + eugenol (2.5%) exhibited both contact and spatial repellency in laboratory assays against this tick species. In spatial repellency bioassays, both nootkatone and the combination of nootkatone + eugenol showed superior results compared to DEET (7%). Moreover, nootkatone and the binary combination (nootkatone + eugenol) displayed high efficacy under field conditions, reinforcing the potential of these compounds in preventing bites from *A. sculptum* nymphs in humans.

Carvacrol and thymol showed mean repellency greater than 90% in laboratory assays after 72 h of evaluation. These monoterpenes are among the most studied botanical compounds for tick control, with proven repellent activity against several species, including *Ixodes ricinus*, *Ixodes scapularis*, *Amblyomma americanum*, *Rhipicephalus microplus*, *Rhipicephalus annulatus*, and *Rhipicephalus sanguineus* sensu lato (s.l.) [14,17,20,21,46]. Jordan et al. [15] also demonstrated the repellent efficacy of carvacrol under field conditions against *A. americanum* and *I. scapularis*. However, despite their potential as botanical repellents for ticks [6], as reinforced by the results of the present study, carvacrol and thymol were not included in the olfactometer or field tests due to certain challenges these molecules may present, such as strong odor, irritant potential [47,48], high volatility, and thermal instability [20,46]. Nevertheless, future studies with these compounds should not be ruled out, as strategies such as nanoencapsulation using nanotechnology may help overcome these limitations [2,49].

Eugenol, tested individually, also demonstrated high contact repellent activity (87%). Predominantly present in the essential oil (EO) of S. aromaticum (clove), this phenylpropanoid exhibits a broad spectrum of biological activities, and its repellent effect has been reported against *R. microplus*, *D. nitens*, *A. sculptum*, *I. scapularis*, and *H. scupense* [27,28,38,50]. In addition to its efficacy, eugenol has pleasant scent and low mammalian toxicity and is even used in dental procedures [51], making it a promising molecule for the development of repellents intended for human use [38].

Nootkatone (5%) showed high contact and spatial repellency, with mean percentages of 92% and 86%, respectively, exceeding the efficacy of DEET (7%). Laboratory and field studies have demonstrated both contact and spatial repellent activity of this sesquiterpene against several tick species, including *R. sanguineus* s.l., *Dermacentor variabilis*, *A. americanum*, *I. scapularis*, and *Ixodes persulcatus* [15,16,52,53,54]. Carr and Salgado [55] suggested that nootkatone temporarily interferes with the thermotaxis of *A. americanum* and *D. variabilis*, preventing ticks from detecting the heat emitted by a host. However, thermotaxis was not evaluated in the present study. Instead, olfactory-based behavioral assays were employed, indicating that this mechanism may contribute to the repellent activity of nootkatone. Further studies are needed to better understand the olfactory physiology/processing of *A. sculptum*, as available data on this subject remain scarce.

In addition to its efficacy, the registration and use of nootkatone as a repellent have also been driven by its biosafety, as this compound is present in foods consumed by humans, and by its odor, which is considered more pleasant than that of DEET [31]. Therefore, nootkatone was selected for the field trials due to its high repellency observed in laboratory assays, along with the growing interest in this molecule.

The combination of nootkatone (2.5%) with eugenol (2.5%) yielded promising results in both laboratory and field assays. The mean contact repellency in the laboratory was 92.8%, with peaks of 100% at specific time points, and 72.2% spatial repellency. In the field study, solutions containing nootkatone and the nootkatone + eugenol combination exhibited repellency rates of 89.1% and 87.6%, respectively, corroborating previous studies on the repellent potential of nootkatone against ticks. Schulze et al. [16] observed 100% repellency of *I. scapularis* and *A. americanum* for up to 14 days on flannel treated with nootkatone (0.76 mg/cm^2^), and 90% repellency of *A. americanum* using CO_2_ traps with flannel treated at 1.0 mg/cm^2^. Jordan et al. [15] reported 100% repellency of adult *I. scapularis* and *A. americanum* using a 10% concentration for 3 and 1 day(s) of evaluation, respectively.

In the laboratory assays, it is noteworthy that both nootkatone and the nootkatone + eugenol combination showed similar contact repellency to DEET and superior performance in the olfactometer test. In a previous study, Barrozo et al. [38] reported 82% repellency under field conditions using a 7% DEET hydroethanolic solution. It is important to note that both studies were conducted in the same location, using the same methodology and during the same season. Comparative studies on the efficacy of nootkatone and DEET against different mosquito species (*Aedes aegypti* and *Aedes albopictus*) have also reported similar repellency levels for both compounds [56,57]. Conversely, using a different methodology, a study on *I. persulcatus* demonstrated that nootkatone outperformed DEET, icaridin, and IR3535 [53]. Given that DEET is widely regarded as the gold standard in arthropod repellency [58], the findings of the present study further support the potential of nootkatone and its combination with eugenol as promising alternatives for the development of tick repellents.

Regarding the nootkatone + eugenol combination, it is important to emphasize that this approach was designed to reduce the concentration of nootkatone without compromising efficacy, given the high cost of this molecule, which may hinder its commercial application [23,25,31,59]. Although there have been advances in the production of nootkatone through liquid fermentation using genetically modified yeasts, the cost this compound remains high [22,23,32,52]. The economic motivation is clear: while 100 mg of nootkatone cost approximately USD 1017, the same amount of eugenol costs around USD 87 [44,45]. Therefore, a 100 mL formulation containing 5% nootkatone would cost approximately USD 50.8, whereas the combination of 2.5% nootkatone + 2.5% eugenol would cost USD 28.6, representing a reduction of nearly 50% (44%).

Another advantage of this combination is the use of a highly volatile compound, such as eugenol, together with a less volatile one with greater residual action, such as nootkatone, which may enhance repellent efficacy. Similar strategies have already been employed to optimize repellent formulations [6,60]. Given the repellent potential of nootkatone and the nootkatone + eugenol combination, future studies focusing on the development of more sophisticated formulations based on nanotechnology and encapsulation are warranted. Recent studies have shown that such approaches can enable controlled release of the active compound, increasing acaricidal activity, thermal and photo-stability, as well as improving solubility while reducing volatility and phytotoxicity [47,61].

## 5. Conclusions

Thymol, carvacrol, nootkatone, and eugenol at 5% concentration, as well as the binary combination of nootkatone + eugenol (2.5% each), showed high efficacy in contact repellency tests against unfed *A. sculptum* nymphs. In spatial repellency assays, nootkatone and its combination with eugenol also demonstrated strong performance, outperforming 7% DEET. Under field conditions, both nootkatone and nootkatone + eugenol maintained high efficacy, with repellency rates above 85% after two hours of exposure. Considering the similar efficacy between solutions (nootkatone and nootkatone + eugenol) and the improved cost-effectiveness, the nootkatone + eugenol combination appears to be the most promising alternative for the development of botanical repellents against *A. sculptum*.

## Figures and Tables

**Table 1:** Repellency percentage (mean ± standard deviation) of unfed *Amblyomma sculptum* nymphs exposed to 5% of thymol, carvacrol, nootkatone, eugenol (5%) and nootkatone + eugenol (2.5%), in a Petri dish assay conducted under laboratory conditions (27 ± 1 °C and 70 ± 10% relative humidity).

Evaluation Time		Control (Ethanol)	Control (Blank)	7% DEET	5% Thymol	5% Carvacrol	5% Nootkatone	5% Eugenol	2.5% Nootkatone + 2.5% Eugenol
Minutes	1	38.3 ± 19.3	46.7 ± 20.5	83.3 * ± 17.6	93.3 * ± 11.7	100.0 * ± 0.0	88.3 * ± 11.2	90.0 * ± 16.1	86.7 * ± 13.1
	15	45.0 ± 17.7	48.3 ± 12.3	80.0 * ± 17.2	93.3 * ± 11.7	91.7 * ± 8.8	95.0 * ± 8.1	86.7 * ± 13.1	90.0 * ± 8.6
	30	56.7 ± 11.7	46.7 ± 15.3	93.3 * ± 11.7	96.7 * ± 7.0	100.0 * ± 0.0	90.0 * ± 11.7	90.0 * ± 11.7	90.0 * ± 11.7
Hours	1	55.0 ± 15.8	53.3 ± 17.2	91.7 * ± 8.8	100.0 * ± 0.0	100.0 * ± 0.0	98.3 * ± 5.3	96.7 * ± 7.0	98.3 * ± 5.3
	2	55.0 ± 19.3	61.7 ± 11.2	90.0 * ± 11.7	100.0 * ± 0.0	98.3 * ± 5.3	93.3 * ± 8.6	93.3 * ± 11.7	100.0 * ± 0.0
	3	55.0 ± 19.3	48.3 ± 14.6	90.0 * ± 11.7	98.3 * ± 5.3	100.0 * ± 0.0	96.7 * ± 7.0	91.7 * ± 8.8	100.0 * ± 0.0
	4	56.7 ± 11.7	51.7 ± 12.3	88.3 * ± 13.7	100.0 * ± 0.0	98.3 * ± 5.3	95.0 * ± 8.1	86.7 * ± 13.1	100.0 * ± 0.0
	24	45.0 ± 24.9	45.0 ± 22.3	77.1 * ± 15.8	81.7 * ± 18.3	95.0 * ± 8.1	95.0 * ± 8.1	81.7 * ± 12.3	88.3 * ± 11.2
	48	41.7 ± 11.8	58.3 ± 22.6	65.0 * ± 12.3	58.3 ± 29.7	80.0 * ± 20.5	93.3 * ± 8.6	81.7 * ± 12.3	91.7 * ± 8.8
	72	55.0 ± 23.6	45.0 ± 11.2	63.3 * ± 13.1	80.0 * ± 15.3	81.7 * ± 14.6	91.7 * ± 8.8	73.3 * ± 19.6	83.3 * ± 15.7
Mean (%) ± standard deviation		50.3 ± 7.0	50.5 ± 5.7	82.2 ± 12.1	90.2 ± 13.4	94.5 ± 7.7	93.7 ± 3.0	87.2 ± 6.8	92.8 ± 6.2

* Repellency was considered significant when the number of ticks on the untreated side of the Petri dish was higher than on the treated side, with a statistically significant difference at the 5% level, according to the chi-square test. Significant difference compared to the control group (*p* < 0.05). DEET: positive control of repellency. Control (ethanol): Petri dish containing one semicircle of filter paper treated with 50% ethanol and one untreated semicircle. Control (blank): Petri dish containing two semicircles of untreated filter paper, with no compounds applied.

**Table 2:** Estimated cost (USD) for producing 100 mL of repellent formulations containing either 5% nootkatone or a combination of 2.5% nootkatone + 2.5% eugenol.

Compound/Combination	Concentration (% m/v)	Amount (g) in 100 mL	Cost per 100 mg (USD)	Estimated Cost (USD)
Nootkatone	5.0	5.0	$1017.00	$50.8
Nootkatone + Eugenol (1:1)	2.5 + 2.5	2.5 + 2.5	$1017.00 + $87.00	$28.6

Cost calculated based on Sigma-Aldrich pricing (accessed on 19 March 2025), considering the production of a 100 mL formulation.

## Data Availability

The original contributions presented in the study are included in the article, further inquiries can be directed to the corresponding author.

## Abbreviations

The following abbreviations are used in this manuscript:

ANOVA	Analysis of variance
CEP	Research ethics committee (Comitê de Ética em Pesquisa)
CEUA	Animal use ethics committee (Comissão de Ética no Uso de Animais)
DEET	N, N-diethyl-3-methylbenzamide
EO(s)	Essential oil(s)
EVZ	School of veterinary medicine and animal science (Escola de Veterinária e Zootecnia)
PPE	Personal protective equipment
RH	Relative humidity
SBP	Brazilian society of parasitology (Sociedade Brasileira de Parasitologia)
SD	Standard deviation
UFG	Federal University of Goiás
